# Sleep Problems in School Aged Children: A Common Process across Internalising and Externalising Behaviours?

**DOI:** 10.3390/clockssleep2010002

**Published:** 2019-12-20

**Authors:** Danielle M. Bayes, Ben Bullock

**Affiliations:** 1 Department of Psychological Sciences, Swinburne University of Technology, Melbourne 3122, Australia; 2 Centre for Mental Health, Swinburne University of Technology, Melbourne 3122, Australia

**Keywords:** sleep problems, children, internalising behaviour, externalising behaviour

## Abstract

Sleep problems are common in childhood and impact cognitive, psychological and physical wellbeing. The current study investigated the association between sleep problems and internalising and externalising behaviour in 114 school-aged children (5–12 years) from four primary schools in Melbourne, Australia. Data were collected using the Sleep Disorder Inventory for Students to measure sleep and the Conners Behavior Rating Scale to assess behaviour, both by parent report. Hierarchical regression analysis, controlling for socioeconomic status and age, identified moderate associations between sleep problems and emotional distress, aggressive behaviour and hyperactivity/impulsivity. Findings suggest screening for sleep problems in children presenting clinically with behavioural issues is a potentially important clinical practice. Additionally, results support the elaboration of transdiagnostic theory, whereby sleep problems are a common process in both internalising and externalising behaviour in children.

## 1. Introduction

Sleep problems are common among children. Between 15%–30% of children experience difficulties with falling and/or staying asleep [1]. This high rate of sleep problems is concerning, as healthy sleep is central to a range of regulatory mechanisms in the human body, and has implications for cognitive, psychological and physical wellbeing [2,3,4]. Investigation of the associations between sleep and behavioural problems in children is necessary for a better understanding of the developmental trajectories that lead to later development of internalising disorders [5] and difficulties with behaviour regulation and impulsivity [6].

The transdiagnostic approach to sleep disorders advanced by Harvey [7], in which sleep disorders are seen as common processes across a range of psychological disorders, has enhanced scientific understanding and management of these disorders, particularly among adolescents. The benefits of implementing a transdiagnostic approach include the use of one powerful intervention rather than discrete interventions for a range of symptoms; less need for practitioners to have competence across a greater range of therapies for varying disorders and symptoms; and a reduced load on carers by only employing targeted strategies for improving sleep. These benefits could conceivably extend to better understanding and management of sleep and psychological problems in primary school-aged children, including both internalising and externalising behaviours in this group.

### 1.1. Sleep Problems and Internalising Behaviours in Children

Sleep problems are commonly associated with internalising symptoms including anxiety, depression, and emotional dysfunction. Children with sleep problems displayed symptoms of generalised anxiety and school-phobia in a cross-sectional study of 79 children aged 8–11 years using self-reports [8]. Dyssomnias, parasomnias and daytime sleepiness were associated with anxiety-related school refusal in a study of 1490 German children aged 8–11 years that used both parent- and self-reports [9]. Additionally, a study of 6–12 year olds from Australia (*n* = 90), assessed using parent-reports of sleep and anxiety and objective measurement of sleep (actigraphy), reported small-to-medium correlations between sleep problems and generalised anxiety symptoms, and small-to-medium correlations between objectively measured variability in sleep schedules and anxiety [10]. Longitudinal research suggests sleep problems during childhood are predictive of later anxiety [5]. The predictive relationship between sleep problems and later depression is less consistent [11,12]. Convergent findings from a relatively smaller number of experimental studies demonstrate causal associations between cumulative sleep restriction and emotion regulation difficulties in children [13], and between acute sleep restriction and both a heightened negative affect and lowered positive affect in adolescents [14].

### 1.2. Sleep Problems and Externalising Behaviours in Children

Sleep problems are also related to externalising behaviour in children. Pesonen et al. [15] found that short sleep duration and other sleep difficulties in 280 children aged eight years old were associated with higher hyperactivity/impulsivity scores from parent-reports. A greater prevalence of parent-reported sleep complaints was reported among children with attention-deficit hyperactivity symptoms (*n* = 79) compared to a non-clinical control group (*n* = 174) [16], whilst sleep quality and quantity in a healthy sample of 7–12 year old children measured by actigraphy was associated with aggressive behaviour and inattention [17]. Furthermore, clinically diagnosed sleep problems that disrupt restful sleep, such as sleep-disordered breathing and obstructive sleep apnoea, are commonly found in children who display inattention, hyperactivity [18], and oppositional behaviours [19], and in children who display aggressive behaviours [20] and conduct problems [21].

Longitudinal research has also reported a relationship between sleep problems and externalising behaviours. A recent one year longitudinal study of 227 Italian children aged 6–10 years found an association between parent-reported sleep problems and teacher-reports of increased hyperactivity and inattentive behaviours, but not conduct problems, at school [22]. A meta-analysis of experimental studies investigating problem sleep, inattention and hyperactivity in youth reported a small-to-medium size relationship between sleep restriction and attention difficulties but not hyperactivity [23]. 

### 1.3. Sleep Problems and Internalising and Externalising Behaviours

A relatively smaller number of studies have explored relationships between sleep problems and both internalising and externalising symptoms. Reduced sleep duration assessed by actigraphy in 6–11 year olds (*n* = 91) was found to be predictive of conduct problems but not hyperactivity or emotional symptoms [24]. In contrast, two large studies, one using parent-reports of sleep problems on the Children’s Sleep Habits Questionnaire (*n* = 912, aged 6–14 years) [25], and one using polysomnography paired with parent-report for insomnia symptoms (*n* = 700, aged 5–12 years) [26] both reported small-to-medium associations with problematic internalising and externalising behaviours. 

Further evidence of an association between sleep problems and both internalising and externalising behaviours comes from a large prospective cohort study conducted in Australia. Quach, Nguyen, Williams, and Sciberras [27] demonstrated that sleep problems were predictive of the later development of internalising behaviours in 3956 children measured five times across a nine year period. A bi-directional longitudinal relationship was also found between sleep problems and externalising behaviours. 

Collectively, the findings of previous studies, using a variety of study designs and spanning multiple age groups and diverse populations, indicate that sleep problems (measured in a variety of ways) are associated with both internalising and externalising behaviours in children. The findings support the notion of sleep as a transdiagnostic process—that is, one in which symptoms are common across more than one disorder [7]. The aim of the present study was to add incrementally to this evidence base by investigating the association between sleep and behavioural problems using clinically-validated assessments of sleep disturbance and internalising and externalising behaviours in children. Consistent with a transdiagnostic approach to understanding the relationship between sleep and these behavioural problems in children, we predicted that greater sleep problems would be associated with higher levels of both internalising and externalising behaviours. Age, sex and socioeconomic status (SES) may influence the relationship between sleep and behavioural symptoms and were also investigated as potential covariates in the current study. 

## 2. Results

### 2.1. Preliminary Analysis

Data screening revealed missing data. Imputation of subscale means was undertaken for cases in which there were <20% missing data. One multivariate and two univariate outliers were deleted and not replaced. Data was evaluated for assumptions of normality, linearity and homoscedasticity by assessing scatterplots of residuals. Some violations of these assumptions were identified (see Section 3.3).

### 2.2. School Characteristics

Table 1 shows the smallest school had the highest survey return rate, while the highest number of surveys were returned from the largest school. The mean return rate (6.5%) was very low compared to other similar studies (e.g., [20]). One-way analysis of variance revealed no significant difference in mean scores between schools for the Sleep Disturbance Index (SDI; *F* (3110) = 1.69, *p* = 0.174) and Conners Behavior Rating Scales(CBRS; ED; *F* (3109) = 0.75, *p* = 0.527; AB; *F* (3110) = 0.20, *p* = 0.897; Hy/Im; *F* (3110) = 0.26, *p* = 0.855).

### 2.3. Participant Characteristics

Table 2 summarises participant characteristics. The sample was equally spread across the sexes, and there was a predominance of nine year olds. The mean age of the children in the sample was 8.40 years (*SD* = 2.12); the range of ages was 5–12 years. Sixty-one percent of the sample reported an annual household income over $100 K, with 41% of this group reporting an income over $200 K. As indicated in Table 3, 80%–89.5% of the sample was in the normal range across age- and sex-adjusted CBRS *T*-score categories and almost all participants scored in the normal range for adjusted SDI (99.1%; *M* = 46.66, *SD* = 5.59). Mean scale scores for the CBRS reflect this trend (AB *M* = 50.67, *SD* = 8.45; Hy/Im *M* = 51.93, *SD* = 11.71), with ED having the highest mean score (*M* = 56.62 *SD* = 14.91). 

Table 4 shows the results of independent sample *t*-tests comparing boys and girls on SDI and CBRS scores (all *p* > 0.05). Pearson correlation analysis, provided in Table 5, showed a weak negative association between age and CBRS-AB and a weak positive association with CBRS-ED. Additionally, a weak negative association was found between annual income and SDI scores. CBRS scales and SDI were all positively correlated.

### 2.4. Sleep Problems and Internalising Behaviour

Table 6 summarises the hierarchical multiple regression used to assess the relationship between sleep problems and internalising behaviour (emotional distress), controlling for annual income and age. Annual income and age were entered at Step 1, explaining 5% of the variance in ED. SDI was entered at Step 2, explaining an additional 13% of the variance in ED, *R^2^_change_* = 0.13, *F_change_* (1, 109) = 17.11, *p* < 0.001. In the final model, two variables were statistically significant independent predictors of ED, with SDI recording a higher beta value (β = 0.37, *p* < 0.001) than age (β = 0.24, *p* < 0.001). SDI explained 12.9% unique variance in ED.

### 2.5. Sleep Problems and Externalising Behaviour

Table 7 and Table 8 summarise the hierarchical multiple regression analyses used to assess the associations between sleep problems and externalising behaviours (AB and Hy/Im, respectively). Annual income and age were entered at Step 1, explaining 6% of the variance in AB. SDI was entered at Step 2 explaining an additional 12% of the variance in AB, *R^2^_change_* = 0.12, *F_change_* (1110) = 16.59, *p* < 0.001. In the final model, one variable was a statistically significant independent predictor of AB, SDI, which explained 12.3% unique variance in AB (β = 0.37, *p* < 0.001).

Annual income was entered at Step 1 of the final hierarchical multiple regression analysis and did not explain significant variance in Hy/Im. SDI was entered at Step 2, explaining an additional 16% of the variance in Hy/Im, *R^2^_change_* = 0.16, *F_change_* (1111) = 21.14, *p* < 0.001. In the final model, one variable was a statistically significant independent predictor of Hy/Im. SDI explained 16% variance in Hy/Im (β = 0.42, *p* < 0.001). 

## 3. Discussion

The results of our investigation support the hypothesis that sleep problems are associated with both internalising and externalising behaviours in primary school-aged children. Regression analyses controlling for any effects of socio-economic status (annual household income) suggested that sleep problems accounted for a statistically significant amount of variance in internalising (emotional distress) and externalising (aggressive behaviour, hyperactivity/impulsivity) behaviours at medium effect sizes, on average.

### 3.1. Sleep Problems and Internalising and Externalising Behaviours

The current study’s findings are broadly consistent with those from previous research. Calhoun et al. [26] found an association between subjectively (parent-report) and objectively (polysomnography) assessed sleep problems and higher scores on internalising and externalising behaviours. Specifically, insomnia symptoms of difficulty initiating and/or maintaining sleep, paired with normal sleep duration were associated with hyperactivity, opposition and inappropriate social behaviour, which, collectively, parallel the aggressive behaviour and hyperactivity/impulsivity assessed by parent-report in the current study. Insomnia symptoms paired with a short duration of sleep were characterised by anxiety and depression in the Calhoun et al. [26] study, similar to the emotional distress assessed by parent-report in our study. Our findings parallel those of Calhoun et al. [26], albeit in the absence of a sleep duration measure. Our findings are also consistent with those of Quach et al. [27], who reported associations between sleep problems and conduct problems, hyperactivity/inattention and emotional problems. The results of this and previous studies therefore show that children experiencing internalising and externalising behaviour also tend to have symptoms of sleep problems. 

Several possible mechanisms underpinning the relationship between sleep and internalising and externalising problems have been proposed. Biological investigations suggest a myriad of possible biological and genetic mechanisms, however none are yet able to elucidate the differential relationship across internalising and externalising behaviours, to our knowledge. Fernandez-Mendoza et al. proposes that a synergistic effect of insomnia symptoms and sleep duration on cortisol levels affects internalising behaviours in the following way: prior to sleep, cortisol levels rise in anxious children [28] and adolescents with depression [29], increasing pre-sleep cognitive and somatic arousal, and thus making it harder to initiate sleep. This insomnogenic response is considered a dysfunction of normal arousal processes driven by the hypothalamic–pituitary–adrenal axis (HPA) [30]. This response in children and adults is associated with a short sleep duration which may explain the relationship with internalising behaviour difficulties.

Conversely, sleep problems associated with externalising behaviours are thought to be driven by an underlying behavioural profile. The insomnia in this case is more akin to the behavioural insomnia of childhood [24] comprising bedtime resistance and learned sleep onset associations (requiring a specific person or item) which the child relies upon to assist in initiating sleep. These issues with initiating and/or maintaining sleep are therefore driven by behaviour, not hyperarousal.

Although the aforementioned mechanistic descriptions are reasonable, they are not the only explanation of the found associations. It is likely that genetic, environmental and familial factors work together to contribute to the associations between sleep problems and internalising and externalising behaviour in children. The findings of the current study tentatively support the hypothesis that sleep problems in school-aged children are a common process in internalising and externalising behaviours. These findings have implications for research, theory, practice and childhood health and wellbeing.

### 3.2. Implications and Future Directions

The current findings build upon transdiagnostic theory by providing supportive evidence of a common association between sleep problems and both internalising and externalising behaviours in children. Nevertheless, much further research is required to elucidate the underlying mechanisms involved in the association between sleep problems and the differing behavioural symptoms. Longitudinal, prospective cohort designs and experimental research designs are required for investigations of the bi-directional nature of these relationships.

In the context of clinical practice, screening children presenting with internalising and externalising symptoms for sleep problems should be part of regular intake procedures. Subsequent identification of a co-morbid sleep problem, either as a maintaining or initiating feature of behavioural problems, can then be appropriately managed. Further study to explore treatment options within a transdiagnostic framework, like that of Harvey’s [7] transdiagnostic intervention for youth sleep and circadian problems, is required. Using one powerful intervention to reduce behaviour severity has the potential to improve a range of health outcomes in children. 

### 3.3. Limitations

The study outcomes should be considered within the context of several limitations. First, generalisability of the results to lower SES populations is uncertain, as our sample comprised a large number of high SES participants. Poorer sleep hygiene practices are more commonly reported in lower SES families compared to higher SES families [31], highlighting the importance of investigations that assess a range of community groups. Second, the exclusive use of parent-report instead of objective methods for the assessment of sleep and behavioural problems introduces potential for rater bias. Although parental involvement in bedtime routines and everyday behaviours is still substantial for school-aged children, future studies should incorporate objective measures of children’s sleep (e.g., actigraphy) and teacher reports of behaviour to further limit the possibility of parent-report rater bias. The return rate of surveys was very low, which could be due to several factors, including the length of the questionnaires, the use of hard copy questionnaires instead of online versions, and the requirement of parent respondents to have at least a secondary school level of English comprehension to successfully complete the instruments. Third, given the clinical nature of the measures used to assess the variables under investigation, a positive skew was expected in the distribution of data, however, a broader range of high scorers on the these variables would have been preferable. Similarly, the heteroscedasticity observed in the distributions of predicted values of the dependent variable against residuals likely indicates that other factors are involved in the relationships under investigation that are worthy of future study. Fourth, previous studies allude to a bi-directional association between sleep and internalising and externalising behaviours [32]. The cross-sectional design of the current study did not permit further exploration of this aspect of the relationship between sleep and behavioural problems. Lastly, the implementation of validation items in the CBRS in the present study was used to aid in screening data, however, future studies should include deeper analysis of these items to provide an overall indication of the validity of participants’ responses. 

It is also worth noting that most participants (99%) reported a SDI *T*-score in the ‘average’ range. Thus, there was little evidence of significant sleep disturbance in this sample. Greater individual variation was observed on the subscales of the SDIS, however the relatively small sample size did not allow for a more fine-grained exploration of these phenomena. Investigation of relationships in a larger sample may yield more variation in SDI score and should be considered in future research, along with analysis of SDIS subscales and their relationship with internalising and externalising behaviours. 

### 3.4. Conclusions

The current findings provide supportive evidence of sleep problems as a common process in both internalising and externalising behaviours. Experimental and longitudinal research is required to consolidate this work and extend it to an assessment of the bi-directional association between sleep and behavioural problems in children. Within the context of the study limitations, the current findings advance transdiagnostic theory as already applied in adult and adolescent settings to school-aged children with comorbid sleep and behavioural problems. By identifying sleep problems early and intervening to improve children’s sleep, substantial gains in health and well-being outcomes in the short and long term are possible. 

## 4. Materials and Methods

### 4.1. Participants

One hundred and fourteen children aged 5–12 years and enrolled in Grades Prep to 6 were recruited from four government schools in Melbourne, Australia. A parent (or guardian) completed a series of questionnaires reporting on their child’s sleep and general behaviour. The study used a cross-sectional design, collecting observational survey data.

### 4.2. Measures

Hard copy surveys consisting of demographic questions and two validated questionnaires were distributed among parent/guardians at each school. 

**Demographic questions**. Twenty seven questions requested information regarding child demographic details. Sex, age and annual income (SES; measure of socio-economic status) were the variables of interest for the current study.

**Sleep Disorders Inventory for Students—Children’s Form (SDIS-C) and Adolescent Form (SDIS-A) [33]**. The SDIS is a screening instrument for paediatric sleep disorders and sleep problems in children, with demonstrated sensitivity (0.82) and specificity (0.91) [33]. The Children’s form, SDIS-C, assesses sleep problems in children aged 2–10 years and the Adolescent form, SDIS-A, is used for adolescents aged 11–18 years. Parents/guardians report sleep problems based on frequency of behaviour over the previous six months. The form contains 30 and 35 (SDIS-C and SDIS-A, respectively) items scored on a 7-point Likert scale ranging from 1 (*never exhibited this behaviour*) to 7 (*always exhibited this behaviour multiple times per hour, daily or nightly*). Higher scores indicate a greater level of sleep disturbance. A total Sleep Disturbance Index (SDI) score ranging from 30–210 is converted to an age- and sex-adjusted *T*-Score with a mean of 50 and standard deviation of 10. *T*-scores can also be used for classification of cases into categories of sleep disturbance (Table 9). Subscales measuring Obstructive Sleep Apnea Syndrome; Periodic Limb Movement Disorder; Excessive Daytime Sleepiness; Delayed Sleep Phase Syndrome; and Narcolepsy (SDIS-A only) are also available.

Validation testing in standardisation samples from both clinical and non-clinical settings show that the SDIS-C has good psychometric properties, including high internal consistency (0.91; 0.90 in the current study), high predictive validity (0.86) and excellent test–retest reliability (0.97) [34]. The SDIS-A, used for a small number of 11 and 12 year-olds in the current study, also has high internal consistency (0.92; 0.90 in the current study), good test–retest reliability (0.86) and high predictive validity (0.96) [34]. Automated scoring software for the SDIS (Child Uplift, 2019) was used to score participant responses.

**Conners Behavior Rating Scale—Parent (CBRS-P) [35]**. The CBRS-P is a 203-item parent-report questionnaire assessing behaviours in children aged 6–18 years. Three of the nine scales were used in the current study to measure internalising and externalising behaviour: (1) emotional distress (ED; worrying, signs of depression and social isolation); (2) defiant/aggressive behaviours (AB; argumentative, poor anger control and/or aggression); and (3) hyperactivity/impulsivity (Hy/Im; high activity levels, problems with impulse control). Behaviours over the past month are scored by parents using a 4-point response scale ranging from 0 (*not true at all*) to 3 (*very much true*). Raw scores range from 0–111 (ED), 0–63 (AB) and 0–33 (Hy/Im), with higher scores indicating greater levels of the measured behaviours. Raw scores are converted to age- and sex- adjusted *T*-Scores with a mean of 50 and standard deviation of 10. Table 10 provides interpretive guidelines for the *T*-Scores. 

The full CBRS-P has strong psychometric properties, including good internal consistency (0.86), and test–retest reliability (0.81) and an acceptable convergent validity [36]. Cronbach’s alpha in the current sample was high for the ED (0.96), Hy/Im (0.86) and AB (0.86) subscales. Automated scoring software for the CBRS (Multi-Health Systems, Toronto, ON, Canada, 2008) was used to score participant responses.

### 4.3. Procedure

Ethical approval for study procedures were provided by the Swinburne University Human Research Ethics Committee and the Victorian Department of Education and Training. Four primary schools were recruited. 

Schools were recruited through contact with School Principals, who provided consent for the school to be involved. Initial expression of interest forms were distributed to all students in three schools and students returning these forms were then provided with a survey pack to be completed and returned to the school. One small school distributed survey packs to all students. Participant recruitment occurred from May to August, during Terms 2 and 3 of the Australian school year.

Survey packs included a plain language statement detailing the time required for participation, possible risks of participation, and issues of confidentiality and privacy. If a parent/guardian agreed to participate in the study they signed a consent form and completed questionnaires as outlined. Once completed, survey packs were sealed in the accompanying envelope and returned to the school within a two week period. Surveys were marked with a unique identification code to ensure anonymity. Parents at each school were provided the opportunity to enter a random draw to receive one of two $50 gift vouchers per school as compensation for their participation in the study. Vouchers were distributed to the random draw winners by mail two weeks after the participation deadline closed at each school.

### 4.4. Statistical Analysis

In addressing study aims, correlations between SES, age, sleep problems and internalising and externalising behaviours were undertaken, as prior research has demonstrated potentially important relationships between these variables [37,38,39]. Independent samples *t*-tests were used to assess sex differences in sleep problems and internalising and externalising behaviours (see, [10]). For hypothesis testing, hierarchical regressions were performed. Sleep problems, measured on the Sleep Disorders Inventory for Students (SDIS), were the main predictor variable. Externalising behaviours (hyperactivity/impulsivity, Hy/Im and aggressive behaviours, AB) and internalising behaviours (emotional distress, ED) were the outcome variables. Annual income, used as a proxy measure of SES, was statistically controlled across all analysis. Given the multiple analyses performed, alpha for assessing statistical significance was set at 0.01 to reduce risk of a Type I error.

## Figures and Tables

**Table 1:** Student Population and Survey Return Rate by School.

School	Student Population *n*	Return Rate *n* (%)	Location ^a^
School A	150	18 (12%)	Northern suburb
School B	440	37 (8.4%)	Eastern suburb
School C	480	20 (4.2%)	Eastern suburb
School D	708	39 (5.6%)	Eastern suburb
Total	1778	114 (6.5%)	

Note: ^a^ Location in relation to Melbourne Central Business District.

**Table 2:** Participant Characteristics by Sex and for the Total Sample.

Characteristic	Girls *n* (%)	Boys *n* (%)	Total *n* (%)
Gender	56 (49.1)	57 (50.0)	113 (99.1) ^a^
Age			
5	2 (3.6)	10 (17.2)	13 (11.4)
6	6 (10.7)	8 (13.8)	14 (12.3)
7	5 (8.9)	10 (17.2)	15 (13.2)
8	8 (14.3)	4 (6.9)	12 (10.5)
9	15 (26.8)	9 (15.5)	23 (20.2)
10	7 (12.5)	5 (8.6)	12 (10.5)
11	8 (14.3)	11 (19.0)	19 (16.7)
12	5 (8.9)	1 (1.7)	6 (5.3)

Note: ^a^ *n* = 1 parent participant chose not to disclose their child’s sex.

**Table 3:** Number of Participants in Each Conners Behavior Rating Scales (CBRS) and Sleep Didorders Inventory for Students (SDIS) *T*-score Category.

	CBRS ^†^			SDIS ^†^	
*T*-Score	ED *n*	AB *n*	Hy/Im *n*	*T*-Score	SDI *n*
>70	17	5	9	>64	
65–69	5	7	6	60–64	1
60–64	13	7	7	<59	113
40–59	77	95	83		
<40	1	0	9		
Total	113	114	114	Total	114

Note: One participant omitted from ED scale analysis across all study analysis due to missing data. ED = Emotional distress; AB = Aggressive behaviour; Hy/Im = Hyperactivity/Impulsivity; SDI = Sleep Disturbance Index. ^†^ See Section 4.2 for interpretation of *T*-score categories for the SDIS and CBRS.

**Table 4:** Independent Sample *t*-tests Comparing Girls and Boys on SDI and CBRS Raw Scale Scores.

Scale	Group	Mean (SD)	*t*-Statistic	*p*-Value
SDIS-SDI	Girls	45.66 (5.64)		
	Boys	47.26 (5.03)	*t* (111) = 0.11	*p* = 0.114
CBRS-ED	Girls	57.00 (15.11)		
	Boys	57.93 (17.40)	*t* (110) = −0.32	*p* = 0.747
CBRS-AB	Girls	50.25 (7.73)		
	Boys	51.59 (10.05)	*t* (111) = −0.10	*p* = 0.920
CBRS-Hy/Im	Girls	51.98 (13.03)		
	Boys	51.57 (10.26)	*t* (111) = −1.31	*p* = 0.192

Note: SDI = Sleep Disturbance Index; ED = Emotional distress; AB = Aggressive behaviour; Hy/Im = Hyperactivity/Impulsivity.

**Table 5:** Correlations Between Age, Annual Income, SDI and CBRS Scales.

	1	2	3	4	5
1. Age	-				
2. Annual income	0.02	-			
3. SDI	−0.06	−0.27 **	-		
4. CBRS-ED	0.21 *	−0.01	0.33 **	-	
5. CBRS-AB	−0.22 *	−0.10	0.32 **	0.30 **	-
6. CBRS-Hy/Im	0.09	0.03	0.38 **	0.43 **	0.36 **

Note: SDI = Sleep Disturbance Index; ED = Emotional distress; AB = Aggressive behaviour; Hy/Im = Hyperactivity/Impulsivity. * *p* < 0.05; ** *p* < 0.01.

**Table 6:** Multiple Regression with Emotional Distress as the Dependent Variable.

	*R*	*R^2^*	*R^2^_Change_*	*B*	*SE*	β	*t*
Step 1	0.22	0.05					
AI				0.06	0.51	0.01	0.12
Age				1.50	0.64	0.22	2.34
Step 2	0.42	0.18 ***	0.13 ***				
AI				0.61	0.50	0.11	1.23
Age				1.64	0.60	0.24 **	2.72
SDI				1.01	0.24	0.37 ***	4.14

Note: AI = Annual income; SDI = Sleep Disturbance Index. ** *p* < 0.01; *** *p* < 0.001.

**Table 7:** Multiple Regression with Aggressive Behaviour as the Dependent Variable.

	*R*	*R^2^*	*R^2^_Change_*	*B*	*SE*	β	t
Step 1	0.25	0.06					
AI				0.32	0.30	0.10	1.08
Age				−0.90	0.37	−0.23	−2.45
Step 2	0.43	0.18 ***	0.12 ***				
AI				0.64	0.29	0.20	2.21
Age				−0.82	0.35	−0.21	−2.38
SDI				0.58	0.14	0.37 ***	4.07

Note: AI = Annual income; SDI = Sleep Disturbance Index. *** *p* < 0.001.

**Table 8:** Multiple Regression with Hyperactivity/Impulsivity as the Dependent Variable.

	*R*	*R^2^*	*R^2^_Change_*	*B*	*SE*	β	*t*
Step 1	0.03	0.00					
AI				0.11	0.42	0.03	0.27
Step 2	0.40	0.16 ***	0.16 ***				
AI				0.61	0.40	0.14	1.52
SDI				0.90	0.20	0.42 ***	4.60

Note: AI = Annual income; SDI = Sleep Disturbance Index. *** *p* < 0.001.

**Table 9:** SDIS *T*-score Classifications [33].

*T*-Score	Guideline
>64	High risk of a sleep disorder (high probability of sleep disorder requiring medical or behaviour intervention)
60–64	Caution range (some sleep problems are exhibited and require monitoring/possible treatment)
≤59	Normal sleep

**Table 10:** CBRS-P *T*-score Interpretative Guidelines [35].

*T*-Score	Standard Deviation (SD)	Guideline
≥70	2 SD above the mean	Very elevated
65–69	1.5–2 SD above the mean	Elevated
60–64	1 SD above the mean	High average
40–59	Average range	Average
<40	1 SD below the mean	Low
